# Potassium Ion Conductivity in the Cubic Labyrinth of a Piezoelectric, Antiferromagnetic Oxoferrate(III) Tellurate(VI)

**DOI:** 10.1002/chem.202102464

**Published:** 2021-09-03

**Authors:** Ralf Albrecht, Markus Hoelzel, Henrik Beccard, Michael Rüsing, Lukas Eng, Thomas Doert, Michael Ruck

**Affiliations:** ^1^ Faculty of Chemistry and Food Chemistry Technische Universität Dresden 01069 Dresden Germany; ^2^ Heinz Maier-Leibnitz Zentrum (MLZ) Technische Universität München Lichtenbergstraße 1 85747 Garching Germany; ^3^ Institute of Applied Physics Technische Universität Dresden 01069 Dresden Germany; ^4^ Max-Planck Institute for Chemical Physics of Solids Nöthnitzer Staße. 40 01187 Dresden Germany

**Keywords:** crystal structures, hydroflux, ion conductivity, oxoferrates, piezoelectric materials

## Abstract

Orange‐colored crystals of the oxoferrate tellurate K_12+6*x*_Fe_6_Te_4−*x*_O_27_ [*x*=0.222(4)] were synthesized in a potassium hydroxide hydroflux with a molar water–base ratio *n*(H_2_O)/*n*(KOH) of 1.5 starting from Fe(NO_3_)_3_ ⋅ 9H_2_O, TeO_2_ and H_2_O_2_ at about 200 °C. By using (NH_4_)_2_TeO_4_ instead of TeO_2_, a fine powder consisting of microcrystalline spheres of K_12+6*x*_Fe_6_Te_4−*x*_O_27_ was obtained. K_12+6*x*_Fe_6_Te_4−*x*_O_27_ crystallizes in the acentric cubic space group *I* $\bar{4}$ 3*d*. [Fe^III^O_5_] pyramids share their apical atoms in [Fe_2_O_9_] groups and two of their edges with [Te^VI^O_6_] octahedra to form an open framework that consists of two loosely connected, but not interpenetrating, chiral networks. The flexibility of the hinged oxometalate network manifests in a piezoelectric response similar to that of LiNbO_3_.The potassium cations are mobile in channels that run along the <111> directions and cross in cavities acting as nodes. The ion conductivity of cold‐pressed pellets of ball‐milled K_12+6*x*_Fe_6_Te_4−*x*_O_27_ is 2.3×10^−4^ S ⋅ cm^−1^ at room temperature. Magnetization measurements and neutron diffraction indicate antiferromagnetic coupling in the [Fe_2_O_9_] groups.

## Graphical abstract

**K(nockout) response**: K_12+6*x*_Fe_6_Te_4−*x*_O_27_ was synthesized under hydroflux conditions. The acentric cubic crystal structure comprises two non‐interpenetrating chiral oxometalate networks formed by edge‐ or corner‐sharing [Te^VI^O_6_] and [Fe^III^O_5_] polyhedra. The potassium ions are mobile in the labyrinth between the networks. The magnetic moments of the iron cations are pairwise antiparallel, and the compound exhibits a strong piezoelectric response.

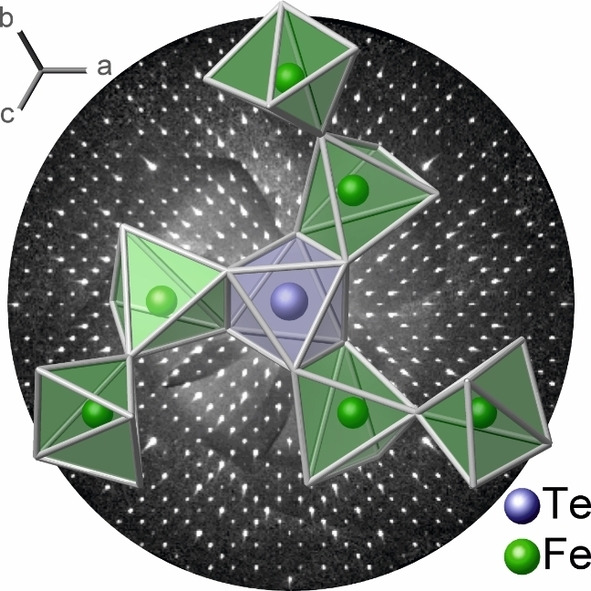

## Introduction

Despite its rarity in the outer layers of the Earth's mantle, tellurium exhibits an extraordinarily rich structural diversity in minerals. Although tellurium is said to be rarer than gold or platinum,^[1]^ tellurium minerals are found about 20 times more frequently than would be expected based on the natural abundance of the element.^[2]^ The cosmic abundance of tellurium is orders of magnitude greater than in the lithosphere,^[3]^ so due to its siderophile nature under high pressure, much of the tellurium is thought to be found in the Earth's core.^[4]^ Several dozen naturally occurring as well as synthetic oxotellurates of abundant metals, such as iron or aluminium, are known.

Oxides with tellurium in oxidation state IV or VI have an overall high structural diversity ranging from isolated oxotellurate polyhedra to three‐dimensional frameworks of connected polyhedra.^[5]^ Both oxidations state are stable under atmospheric conditions and can therefore occur simultaneously in one compound. An analysis of 100 tellurates(VI) with sixfold coordination showed only minor deviations from the average bond length Te^VI^−O=192(4) pm, while in the 66 tellurates(IV) reviewed the coordination splits into three short Te^IV^−O=191±8 pm and three long distances Te^IV^−O=285±40 pm.^[6]^ The latter is usually attributed to a stereoactive lone pair. Various reviews report about the crystal chemistry of tellurates,[^7^, ^8^, ^9^] with a recent paper discussing characteristic structural features in detail.^[5]^

Iron(III) and tellurium(VI) differ considerably in several properties, for instance, coordination behavior by oxygen, presence of unpaired *d* electrons and hence magnetism and color, and also in natural abundance, iron having overwhelming availability on Earth. The combination of iron(III) with alkali metals (*A*) yields a large number of oxoferrates(III), for instance K_5_FeO_4_,^[10]^ K_6_Fe_2_O_6,_ ^[11]^ K_14_Fe_4_O_13_,^[12]^ *A*_4_Fe_2_O_5_ (*A*=Na, K)^[13]^ or *A*FeO_2_ (*A*=K, Rb, Cs),^[14]^ which represent only a small fraction of a large variety. The examples listed are ordered by increasing connectivity, starting with isolated [FeO_4_] tetrahedra, proceeding to tetrahedra pairs and small chain fragments of ferrate(III) tetrahedra, and ending with two‐ and three‐dimensional networks. With increasing complexity, the *A*/Fe ratio tends to decrease. The iron atoms in potassium ferrates(III) exhibit predominantly tetrahedral coordination, but [FeO_6_] octahedra also exist, as in the β‐aluminate type K_1+*x*_Fe_11_O_17_.^[15]^ Iron(III) is known for its versatile coordination behavior with oxygen, which includes the rare trigonal‐bipyramidal coordination observed in FeVO_4_.^[16]^

A few structures of alkali‐metal tellurates(VI) are known with structural motifs that differ from the typical rigid coordination behavior of tellurium(VI). These structures include isolated [TeO_6_] octahedra in Li_4_TeO_5_,^[17]^ isolated tetrahedra in combination with trigonal bipyramids in Rb_6_Te_2_O_9_,^[18]^ helices of edge‐sharing [TeO_6_] octahedra in Na_2_TeO_4_,^[19]^ or even a three‐dimensional network consisting of corner‐sharing [TeO_6_] octahedra in Na_2_Te_2_O_7_.^[20]^

Four different compounds are known in the alkali‐metal ferrate(III) tellurates(VI) group: the garnet Na_3_Te_2_[(Fe,Al)O_4_],^[21]^ the solid electrolyte Na_2_LiFeTeO_6_ with alternating edge‐sharing ferrate(III) and tellurate(VI) octahedra,^[22]^ the lithium‐rich Li_3+1.5*x*_Fe_3−2.5*x*_Te_*x*_O_6_ (0.1≤*x*≤1.0)^[23]^ with a rock salt superstructure, and the defect pyrochlore KFe_0.33_Te_1.67_O_6_.^[24]^ In all these structures, the [Te^VI^O_6_] octahedra are not directly connected.

Here, were we report the hydroflux synthesis of the new potassium ferrate(III) tellurate(VI) K_12+6*x*_Fe_6_Te_4−*x*_O_27_, its crystal structure, chemical and thermal stability, magnetic and piezoelectric properties, and ion conductivity.

## Results and Discussion

### Synthesis

The hydroflux method, in which an approximately equimolar mixture of water and alkali metal hydroxide is used as the reaction medium, represents an intermediate route between hydrothermal and flux synthesis, taking into account water content, melting point and boiling point.^[25]^ The properties of the hydroflux medium are similar to those of a hydroxide melt, such as the strongly basic character and thus high solubility for oxidic compounds. In addition, the hydroflux synthesis generates excellent yields of well‐crystallized products, including single crystals suitable for structure analysis.^[26]^ However, numerous reaction parameters affect product formation, which must be optimized for each class of compounds. These parameters include the choice of starting materials, their ratio, base concentration, fill level of the reaction vessel, temperature, and washing procedure. Nevertheless, the hydroflux approach represents a simple, fast, pressureless, resource‐efficient, and highly versatile synthesis method for oxides and hydroxides with high crystallinity.^[27]^

**Figure 1:**
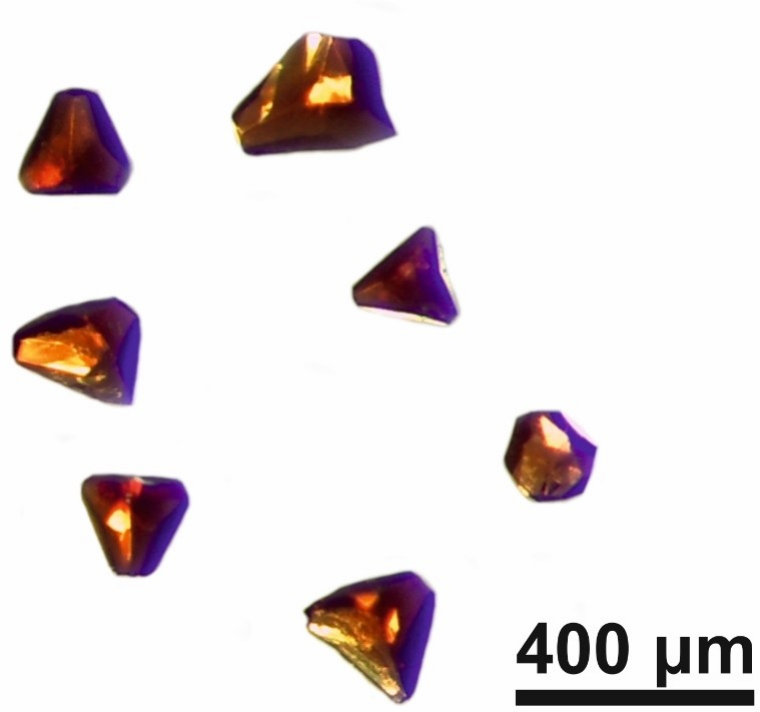
Photograph of selected K_12+6*x*_Fe_6_Te_4−*x*_O_27_ crystals.

Orange‐colored crystals of the potassium hexaferrate(III) tetratellurate(VI) K_12+6*x*_Fe_6_Te_4−*x*_O_27_ with the characteristic shape of triakis tetrahedra were obtained in quantitative yield (based on iron) from a potassium hydroxide hydroflux with *q*(K)=*n*(H_2_O)/*n*(KOH)=1.5 starting from Fe(NO_3_)_3_ ⋅ 9H_2_O, TeO_2_ and H_2_O_2_ (Figure 1). Following a long‐known procedure to synthesize tellurates(VI),^[28]^ TeO_2_ was dissolved in the reaction medium and oxidized with H_2_O_2_. To this mixture, Fe(NO_3_)_3_ ⋅ 9H_2_O was cautiously added in small portions, since iron(III) catalyzes the decomposition of residual H_2_O_2_ in a vigorous reaction.^[29]^ After sealing the autoclave, the mixture was reacted at 200 °C for 10 h and then slowly cooled down to room temperature. We used an excess of the tellurium source (ca. 10 %) to prevent the formation of by‐products such as K_2−*x*_Fe_4_O_7−*x*_(OH)_*x*_ or K_2_Fe_2_O_3_(OH)_2_, which were previously synthesized under similar reaction conditions but without TeO_2_.[^30^, ^31^] The unreacted tellurates(IV/VI) were removed together with the hydroflux medium by washing with water. Powder X‐ray diffraction confirmed that the orange‐colored product was single‐phase K_12+6*x*_Fe_6_Te_4−*x*_O_27_ (Figure S1 in the Supporting Information).

When H_2_O_2_ was exchanged with water, the main product formed was the ferrate K_2−*x*_Fe_4_O_7−*x*_(OH)_*x*_, although some crystals of K_12+6*x*_Fe_6_Te_4−*x*_O_27_ with sizes up to 0.5 mm were still formed (Figure S2). The oxidizing agent probably was molecular oxygen, which was trapped when the reaction mixture was prepared under laboratory conditions. According to Lux et al., molecular oxygen and molten alkali‐metal hydroxides form peroxide and superoxide anions [Eqs. (1) and (2)].^[32]^ We suspect that these species are also present in an aqueous hydroflux system.

(1) $$4\;\mathrm{OH}{}^{-}+O{}_{2}\vec{\leftarrow }2\;O{}_{2}{}^{2-}+2\;H{}_{2}O\;\left(\mathrm{peroxide}\;\mathrm{formation}\right)$$

(2) $$O{}_{2}{}^{2-}+O{}_{2}\vec{\leftarrow }2\;O{}_{2}{}^{-}\;\left(\mathrm{superoxide}\;\mathrm{formation}\right)$$

To further investigate the formation of K_12+6*x*_Fe_6_Te_4−*x*_O_27_ under hydroflux conditions, we studied the system with TeO_2_ and Fe(NO_3_)_3_ ⋅ 9H_2_O at different water‐base ratios *q*(K) using the same reaction parameters as described above. Experiments with *q*(K) between 1 and 3 led to the formation of single‐phase K_12+6*x*_Fe_6_Te_4−*x*_O_27_ (Figure S3). At *q*(K)=4.0, K_12+6*x*_Fe_6_Te_4−*x*_O_27_ is still the main phase, but few weak reflections of an unknown side‐phase were visible in the PRXD pattern of the washed product. At even higher water/base ratios, the K_12+6*x*_Fe_6_Te_4−*x*_O_27_ crystals tended to be larger but frequently intergrown (Figure S4). For *q*(K)≥10, the diffraction pattern of the products showed reflections of β‐FeOOH only (Figure S5).

The use of (NH_4_)_2_TeO_4_ instead of TeO_2_/H_2_O_2_ as starting material with otherwise unchanged reaction parameters resulted in a fine orange‐colored powder of K_12+6*x*_Fe_6_Te_4−*x*_O_27_ composed of microcrystalline spheres, as evidenced by scanning electron microscopy (SEM; Figure S6). These spheres appear to be hollow, as each visible broken or fragmented sphere has cavities. This suggests rapid crystallization favored by the high availability of tellurium(VI) and possibly a template effect of the ammonium cations.

K_12+6*x*_Fe_6_Te_4−*x*_O_27_ is sensitive to water and humid air and should therefore be stored in a dry atmosphere. After some crystals had been stored under ambient conditions for a week, a precipitate had formed on their surface. After several weeks in air, white crystallites had grown on the orange‐colored crystals (Figure S7). EDX measurements on these white crystals showed high levels of potassium, oxygen and carbon, suggesting the formation of potassium carbonate. Similar observations had been reported for K_2−*x*_Fe_4_O_7−*x*_(OH)_*x*_. Although the larger crystals of K_12+6*x*_Fe_6_Te_4−*x*_O_27_ can be washed with water without visible decomposition phenomena on their surfaces (Figure S8), the large surface of fine powder obtained from (NH_4_)_2_TeO_4_ clearly promoted decomposition. In this case, we had to use methanol instead of water for washing, otherwise an amorphous product was obtained.

### Crystal structure

**Figure 2:**
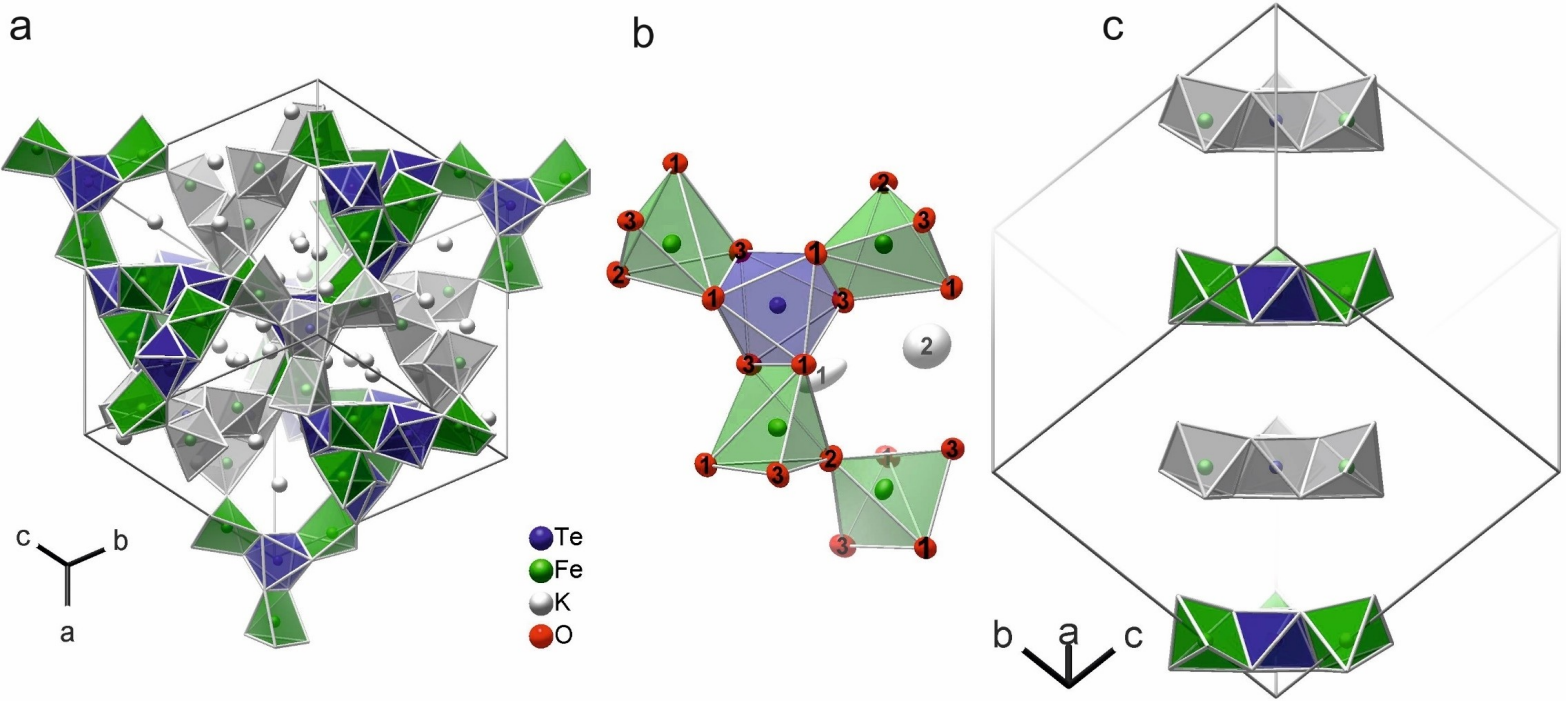
a) Crystal structure of K_12+6*x*_Fe_6_Te_4−*x*_O_27_ projected approximately along [111]. The partially occupied potassium K2 positions are omitted for clarity. Colored and gray‐scale polyhedra show oxometalate frameworks of opposite chirality. b) Detail of the structure with ellipsoids that enclose 95 % of the probability density of the atoms at 100(1) K. c) Sequence along the [111] direction of propeller‐like [TeFe_3_O_15_] fragments, which alternate in their chirality. The orientation of the groups complies with the absence of inversion symmetry and a polar threefold axis.

X‐ray diffraction on a single‐crystal of K_12+6*x*_Fe_6_Te_4−*x*_O_27_ revealed a cubic structure with the acentric space group I$\bar{4}$ 3*d* (no. 220) and the lattice parameter *a*=1474.4(1) pm at 100(1) K (Figure 2, Tables S1–S3). K_12+6*x*_Fe_6_Te_4−*x*_O_27_ adopts a new structure type. We found no match in the inorganic crystal structure database ICSD with respect to the space group, the cell parameter and the Wyckoff sequence.^[33]^

The nonstoichiometry was refined to *x*=0.222(4), which corresponds to K_13.33(2)_Fe_6_Te_3.778(4)_O_27_ (for *Z*=4). Besides the fully occupied position K1, there is the partially occupied, disordered site K2, whose charge is compensated, surprisingly not by reduction to iron(II) or tellurium(IV), but by a corresponding deficiency of tellurium(VI). The refined composition agrees with the results of an energy‐dispersive X‐ray (EDX) analysis within the specified accuracy (Table S4).

In the crystal structure, pairs of [Fe^III^O_5_] square pyramids share an apical oxygen atom in linear [Fe_2_O_9_] groups (Figure 2a,b). Edge‐sharing with [Te^VI^O_6_] octahedra forms a complex oxometalate framework. The propeller‐like arrangement of three [FeO_5_] pyramids around each [TeO_6_] octahedron introduces chirality (Figure 2b). The structure consists of two non‐interpenetrating 3D oxometalate frameworks with opposite chirality (Figure 2c). The orientation of the characteristic [TeFe_3_O_15_] propeller fragments complies with the absence of inversion symmetry and polar threefold axes along the <111> directions. The remarkable observation that none of the investigated crystals was an inversion twin can be rationalized by the 3D connectivity of the structure. The two frameworks are interconnected by the *spiro* oxygen atoms of the [Fe_2_O_9_] groups (Figure S9). The network connectivity is three for the [TeO_6_] as well as for the [FeO_5_] nodes. The potassium cations are located in the continuous three‐dimensional labyrinth between the oxometalate polyhedra (Figure 2a). The graphical representation of the structure gives the impression of an open framework. In fact, K_12+6*x*_Fe_6_Te_4−*x*_O_27_ has a density of only 3.67 g cm^−3^, which is about 5 % lower than the weighted densities of its constituents [K_12_Fe_6_Te_4_O_27_=6 K_2_O ⋅ 3 Fe_2_O_3_ ⋅ 4 TeO_3_; (6 ⋅ 2.35+3 ⋅ 5.24+4 ⋅ 5.07) g cm^–3^ / 13=3.85 g cm^–3^].

A more detailed view reveals that the square pyramid around the iron(III) atom (Wyckoff position 24*d*, site symmetry 2.) is distorted. The Fe−O bond lengths to oxygen atoms O1 and O2 of the base are 198.4(1) and 203.3(1) pm, and, thus, longer than to the apical O3 with 185.1(1) pm. The basis of the [FeO_5_] pyramid is bent by about 16°. The angle Fe−O3−Fe in the [Fe_2_O_9_] group is 180 by symmetry (O3 on 12*a*, $\bar{4}$ ). Only a few inorganic oxides that exhibit iron atoms with square pyramidal coordination are known. One example is FeVO_4_,^[16]^ whose Fe−O bond lengths are rather similar to those in K_12+6*x*_Fe_6_Te_4−*x*_O_27_.

The octahedron around the tellurium(VI) atom (Wyckoff position 16*c*, site symmetry .3.) is slightly distorted. The two Te−O distances of 193.4(1) and 193.7(1) pm differ by less than 1 % from the average Te−O bond length of 192(4) pm as discussed by Mills and Christy.^[6]^ The O−Te−O *trans*‐angle is 171.3(1). The three symmetry‐equivalent edges of the [TeO_6_] octahedron that connect to the neighboring iron atoms are more than 20 pm shorter than the others.

The bond valence sums[^34^, ^35^] for iron *ν*(Fe)=∑exp[(*R_ij_*−*d_ij_*)/37 pm)]=2.82 and tellurium *ν*(Te)=5.53 are slightly smaller than expected for iron(III) and tellurium(VI), but nonetheless support the assigned oxidation states. In the case of tellurium, the deviation might be due to the 5.5 % unoccupied positions.

**Figure 3:**
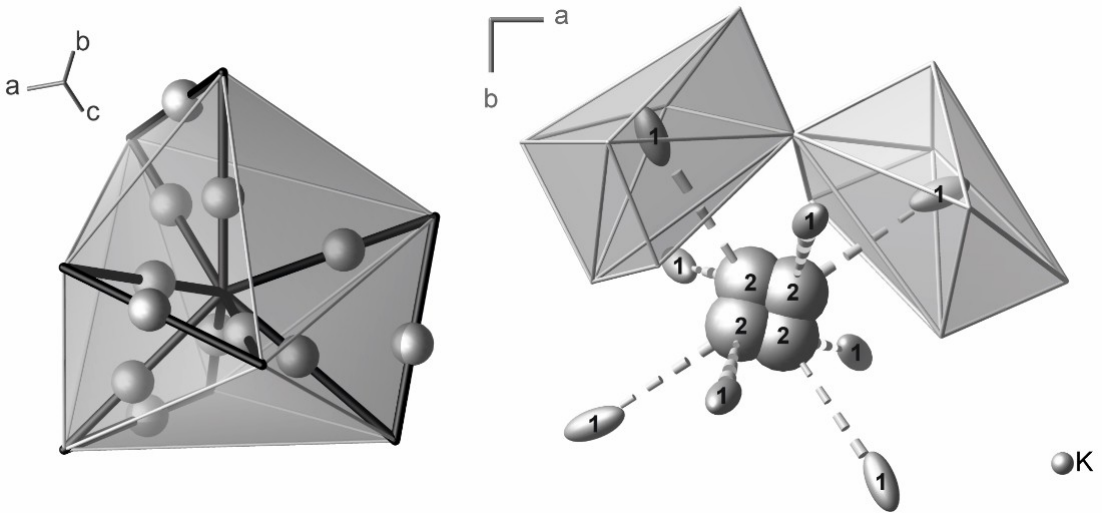
Left: Black lines connect Wyckoff positions 12*b* (each centering four nearby K2 positions) and represent the potassium transport channels. The K1 atom (shown as a sphere) is located in the middle between two of these nodes. Right: Detail of the potassium transport paths, which corresponds to the center of the representation on the left side. Ellipsoids enclose 95 % of the probability density of the atoms at 100(1) K.

The potassium atom K1 – that is, the fully occupied potassium position (48*e*, site symmetry 1) – is coordinated by seven oxygen atoms forming a distorted capped trigonal prism with K−O distances ranging from 267.2(1)–308.0(1) pm (Figure 3, left). This polyhedron is rather flat and has wide trigonal bases. The O⋅⋅⋅O distances within the bases [416.9(4) pm on average] are about 50 % longer than those in the edges on the sides [280.8(4) pm on average]. K1 shows an elongated displacement ellipsoid, which points towards the trigonal bases and the large cavity directly behind them (Figure S9). The atoms K2 are disordered on four partially occupied positions (48*e*, site symmetry 1) close to the special position 12*b* with site symmetry $\bar{4}$ . Their occupancy of 11.1(2) % corresponds with the K_6*x*_=K_1.33_ fraction in the sum formula. The atom K2 is surrounded by eight oxygen atoms forming a strongly distorted double capped trigonal prism with four short K−O distances [298(1) pm on average] and four longer ones at the opposite site [359(1) pm on average].

The Wyckoff site 12*b* is the center of the large cavity in which the channels intersect. The labyrinth of channels (including its oxometalate walls) can thus be visualized by connecting the sites 12*b* (Figure 3, right). As can be expected for the crossing point of four (undulated) <111> channels, the site 12*b* has a connectivity of eight. The associated virtual polyhedron with eight vertices is a bisdisphenoid (triangular dodecahedron). The K1 atom lies exactly on the connecting line between two nodes. Thus, the structural prerequisites for potassium cation conductivity are suboptimal. Despite the favorable 3D labyrinth, its flexibility and the apparently large cross sections within the channels, the energetically stable and fully occupied K1 position is disadvantageous, because it hinders mobility along the channels.

### Chemical and thermal stability

K_12+6*x*_Fe_6_Te_4−*x*_O_27_ slowly hydrolyses in moist air under protonation of oxide ions to hydroxide ions and segregation of potassium hydroxide. To characterize the influence of this mass transport on the crystal structure, the diffraction pattern of a single‐crystal was measured once a week until complete decomposition. The experiments were performed at room temperature to avoid thermal stress. Between the measurements, the crystal was stored in a Petri dish with a humid atmosphere (close to 100 % humidity) to accelerate the hydrolysis process. Detailed information on the crystal structure is given in Table S5. After six weeks, the crystal was considered to be completely decomposed as no more Bragg reflections could be detected. Including the measurement of the pristine crystal, six diffraction experiments were performed. In the structure refinements of the aged crystal, the tellurium content of the pristine crystal was fixed. The hydrogen atoms of the hydroxide groups, which are necessary for charge balance, could not be located in the diffraction experiments.

**Figure 4:**
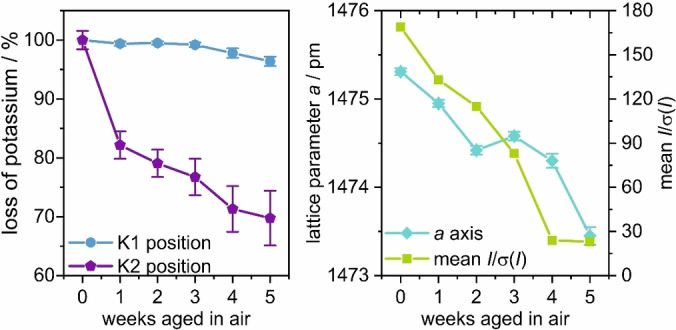
Decomposition of a K_12+6*x*_Fe_6_Te_4−*x*_O_27_ single crystal in moist air over a period of five weeks. Left: Loss of potassium on the positions K1 and K2 relative to the pristine crystal. Right: Development of the lattice parameter and the mean diffraction intensity *I*/*σ*(*I*).

In the course of hydrolysis, the potassium content of both positions K1 and K2 decreased (Figure 4). Whereas the depopulation of the K1 position remained below 5 %, the K2 position lost more than 30 % of its initial potassium content. On the absolute scale, both positions lost approximately the same amount of potassium. The chemical composition of the five weeks aged crystal (5w) was refined to K_12.7(1)_Fe_6_Te_3.74_O_26.1(1)_(OH)_0.9(1)_ (*x*=0.11), evidencing a potassium loss of 7 % as compared to the initial composition K_13.6(1)_Fe_6_Te_3.74(1)_O_27_ (*x*=0.27). The lattice parameter contracted by only 0.2 % before the break‐down of the crystalline long‐range order. In particular, the K1 atoms, which fix the positions of the two flexible oxometalate frameworks against each other, seem to be essential for the stability of the structure.

The thermal stability of K_12+6*x*_Fe_6_Te_4−*x*_O_27_ in air was investigated by annealing samples at various temperatures in an open crucible for one day. All solid products were characterized by powder X‐ray diffraction (PXRD) at room temperature (Figure S10). Up to 700 °C, the diffraction pattern of K_12+6*x*_Fe_6_Te_4−*x*_O_27_ remained essentially unchanged, despite a slight shift in reflection positions. Fits of the powder patterns with the Le Bail method^[36]^ revealed that the lattice parameter decreases with increasing annealing temperatures, reaching a minimum at 500 °C, where *a* is 0.4 % shorter than that of the untreated sample (Figure S11). Segregation of K_2_O should be responsible for this effect, which has also been reported for K_2−*x*_Fe_4_O_7−*x*_(OH)_*x*_.^[30]^ K_2_O is thermodynamically more stable than other potassium oxo‐compounds under these conditions,^[37]^ and it is expected to sublime or react with the Al_2_O_3_ crucible to form KAlO_2_.[^38^, ^39^] Higher annealing temperatures led to a slight increase in the lattice parameter following thermal expansion. Thermal treatment at 800 °C led to the decomposition of the compound and the formation of the potassium‐poor oxoferrates K_1.8_Fe_10.7_O_17_ and KFeO_2_ (Figure S12).^[14]^ We assume that the TeO_3_ moiety evaporated by decomposition to O_2_ and TeO_2_ (partial pressure ∼1 mbar at 800 °C).^[40]^

### Potassium ion conductivity

**Figure 5:**
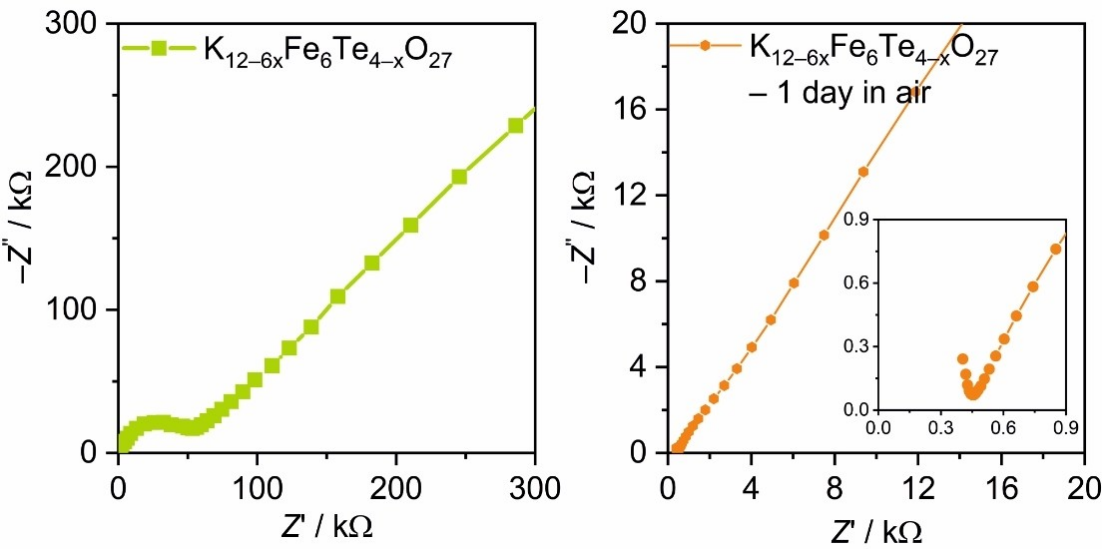
Nyquist plots of impedance measurements recorded at room temperature on cold‐pressed pellets from K_12+6*x*_Fe_6_Te_4−*x*_O_27_ directly after the grounding (left) and after storing one day in air (right).

Crystals of K_12+6*x*_Fe_6_Te_4−*x*_O_27_ were ground to a fine powder using a ball mill and then cold‐pressed into pellets, which were analyzed by electrochemical impedance spectroscopy (EIS) either immediately after preparation (to minimize decomposition effects) or after prior annealing in air at 500 °C for 12 h. Potassium ion conductivity was analyzed in a frequency range between 1 MHz and 0.1 Hz. The recorded impedance is displayed in Nyquist plots in Figures 5, S13 and S14. As expected for an ion conductor, a semicircle and a linear part are present in the Nyquist plot. The linear part is caused by Warburg diffusion and the semicircle results from the capacitance and ohmic resistance of the sample. For some samples, induction effects cause distorted semicircles. The ion conductivity calculated from the impedance can be found in Table S6.

**Figure 6:**
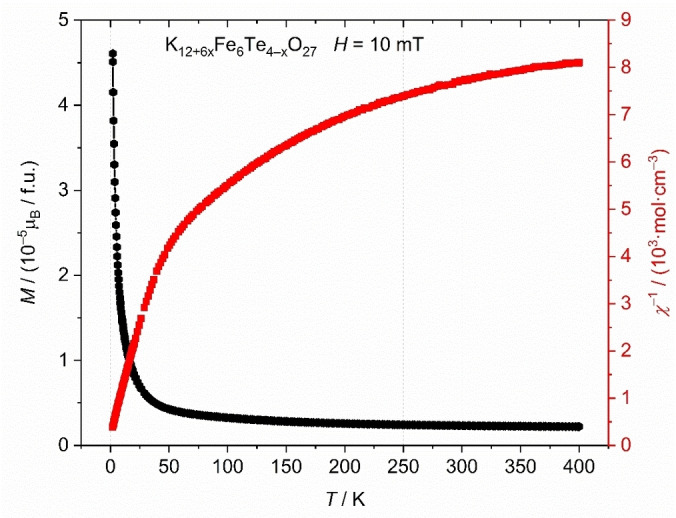
Magnetic susceptibility of K_12+6*x*_Fe_6_Te_4−*x*_O_27_ in an external magnetic field of 10 mT.

The ion conductivity of K_12+6*x*_Fe_6_Te_4−*x*_O_27_ measured immediately after ball‐milling is quite low at 2.3×10^−6^ S ⋅ cm^−1^ (Figure 6). Storing the powder in air for one day greatly increased the ion conductivity to 1.7×10^−4^ S ⋅ cm^−1^. After one week in air, a further slight increase of the ion conductivity was observed (2.3×10^−4^ S ⋅ cm^−1^, Figure S15). As mentioned earlier, KOH segregates from K_12+6*x*_Fe_6_Te_4−*x*_O_27_ when stored under ambient conditions, which is accelerated by annealing the sample. The shorter diffusion path and the larger surface created by grinding cause faster segregation and wetting of the solid by the hygroscopic KOH. Therefore, we assume that the ion conductivity of the aged samples is mainly determined by the mobility of the potassium cations in the liquid phase. A similar phenomenon was observed for the oxohydroxoferrates K_2−*x*_(Fe,*M*)_4_O_7−*y*_(OH)_*y*_ (*M*=Si, Ge, Ti, Mn, Ir).^[41]^ After drying the aged sample at 500 °C in air for 12 h, the ion conductivity was only 1.2×10^−6^ S cm^−1^. In another experiment, the segregated potassium hydroxide of an aged sample was removed by washing with methanol, which resulted in a similar reduction of the ion conductivity (10^−7^ S cm^−1^, Figure S16). According to PXRD, the washing process did not cause decomposition of K_12+6*x*_Fe_6_Te_4−*x*_O_27_ (Figure S15). Annealing the methanol‐washed sample in air at 500 °C did not change its ion conductivity. The ion conductivity of the microcrystalline powder obtained by a synthesis starting from (NH_4_)_2_TeO_4_ was similar to that of the ball‐milled powder.

### Piezoelectricity

The combination of an acentric space group, a rather open framework structure, the structural flexibility that is introduced by edge‐ and corner‐sharing of coordination polyhedra, and highly charged cations motivated us to analyze the local piezoelectric response of a selected K_12+6*x*_Fe_6_Te_4−*x*_O_27_ crystal by piezo‐response force microscopy (PFM). Two types of measurements were performed. First, the piezoelectric amplitude was analyzed as a function of frequency between 25 and 500 kHz and at a constant amplitude. Second, the amplitude of the piezo response was measured as a function of drive voltage at a constant frequency of 175 kHz, where no spectral feature was apparent. The piezoelectric response of K_12+6*x*_Fe_6_Te_4−*x*_O_27_ is comparable to the one of LiNbO_3_ (Figure S16). An approximately linear increase of the piezoelectric response with the voltage was observed. At about 6 V, however, the piezoelectric amplitude decreased abruptly before continuing to increase linearly, which could be caused by an onset of K1 cation mobility. Besides the *spiro*‐oxygen atom in the center of the [Fe_2_O_9_] group, the potassium cations are the only structural element that connect the two independent oxometalate networks. Their mobility could allow for additional modes and static deformation of structure and thus a stronger piezoelectric effect.

### Magnetization and magnetic structure

The temperature dependence of the magnetic susceptibility of K_12+6*x*_Fe_6_Te_4−*x*_O_27_ was determined between 2 and 400 K in an external field of *μ*_0_ *H*=10 mT (Figure 6). The compound is basically nonmagnetic with a magnetic moment of 3.3×10^−6^ μ_B_ per formula unit at 100 K. The increase of the magnetic susceptibility below 30 K is probably due to a paramagnetic impurity. The small value suggests full compensation of the individual iron(III) moments by an antiparallel alignment within the [Fe_2_O_9_] groups. This matches the Goodenough‐Kanamori‐Anderson rules,[^42^, ^43^, ^44^] which postulate an antiferromagnetic coupling if the Fe−O−Fe angle is close to 180.

Neutron diffraction data were collected for a powder sample of K_12+6*x*_Fe_6_Te_4−*x*_O_27_ at 300 and 4 K (Figure S17). The intensities and positions of the reflections are very similar at the two temperatures [*a*(4 K)=1469.03 pm; *a*(300 K)=1473.95 pm]. No magnetic scattering contributions were found in the neutron scattering data, and refining magnetic moments on the iron atoms resulted in unreasonable fits, even without any symmetry restrictions. The absence of long‐range magnetic order can be rationalized by the spatial separation of the [Fe_2_O_9_] groups [Fe⋅⋅⋅Fe 520.7(1) pm] and the lack of a superexchange path across the [TeO_6_] octahedra. Moreover, it can be deduced that the magnetic moments cannot be aligned parallel to the Fe−O−Fe axis, as in this case there would be long‐range order by space‐group symmetry. An inclined (probably orthogonal) orientation of the moments, however, does not match the two‐fold axis along the Fe−O−Fe sequence and leads to an uncorrelated orientation of the spin pairs in the [Fe_2_O_9_] groups and, thus, to the observed lack of magnetic Bragg reflections.

Hence, the neutron powder diffractograms were fitted by considering the nuclear structure only. The Rietveld refinements confirmed the SC‐XRD results concerning potassium disorder, elongated displacement ellipsoid for the K1 atom and occupancies of the Te and K2 positions (Figures S18 and S19). Some weak additional reflections of an unknown impurity phase were found in the neutron powder data; some of them at high diffraction angles, which make an additional magnetic contribution of K_12+6*x*_Fe_6_Te_4−*x*_O_27_ unlikely.

## Conclusions

K_12+6*x*_Fe_6_Te_4−*x*_O_27_ was synthesized in a potassium hydroxide hydroflux at about 200 °C. It forms at a water/base ratio of 1≤*q*(K)≤3. At *q*(K)=3, intergrown crystals with sizes of up to about 1 mm were obtained. Lower base concentrations led to the formation of β‐FeOOH. The particle size and morphology of K_12+6*x*_Fe_6_Te_4−*x*_O_27_ can be influenced by the choice of the tellurium source. K_12+6*x*_Fe_6_Te_4−*x*_O_27_ has a non‐centrosymmetric cubic structure consisting of two non‐interpenetrating chiral frameworks formed by edge‐ and vertex‐sharing [TeO_6_] and [FeO_5_] polyhedra. The latter are connected into [Fe_2_O_9_] groups, in which the magnetic moments of the iron(III) cations are coupled in an antiparallel manner. The comparatively low density of the compound and the hinged polyhedral framework cause a substantial piezoelectric response. The labyrinth between the two frameworks accommodates the potassium cations, which show only moderate mobility at room temperature until the compound partially hydrolyses. Only about 7 % reduction in the potassium content due to leaching of KOH is tolerated before the structure collapses. Upon heating in air, K_12+6*x*_Fe_6_Te_4−*x*_O_27_ loses some K_2_O but is essentially stable up to 700 °C.

## Experimental Section

**Synthesis**: Single‐crystals of K_12+6*x*_Fe_6_Te_4−*x*_O_27_ were synthesized in a KOH (86 %, Fisher Scientific) hydroflux with a water/base ratio of 1.5 : 1. This reaction was carried out in a PTFE‐lined 50 mL Berghof type DAB‐2 autoclave to prevent water loss. Starting from 0.5 mmol TeO_2_ (99.9 %, abcr), 1 ml H_2_O_2_ (30 %, Fischer Scientific) and 2 ml deionized water, 6.7 g KOH were added in small portions. After the oxidation of TeO_2_ finished, one small crystal of Fe(NO_3_)_3_ ⋅ 9H_2_O (≥98 %, Sigma−Aldrich) was added to the mixture. When the violent reaction had finished, the remaining 0.75 mmol of Fe(NO_3_)_3_ ⋅ 9H_2_O were added. The autoclave was heated to 200 °C at 2 K ⋅ min^−1^, held for 24 h before being cooled down at a rate of 0.5 K ⋅ min^−1^ to room temperature. The orange‐colored crystals were isolated by washing with water and stored under argon.

For neutron diffraction experiments, 7 g of K_12+6*x*_Fe_6_Te_4−*x*_O_27_ were synthesized in a PTFE‐lined 250 mL Berghof type HR‐200 autoclave. A KOH hydroflux with a water‐base ratio of *q*(K)=1.3 was used. The starting materials Fe(NO_3_)_3_ ⋅ 9H_2_O (25 mmol) and (NH_4_)_2_TeO_4_ (17 mmol, 99.5 %, abcr) were dissolved/suspended in 55 ml of deionized water. The potassium hydroxide had to be added slowly in 20 g portions to avoid boiling. Additionally, the PTFE inlet was cooled with cold water. The autoclave was heated to 200 °C at 2 K ⋅ min^−1^, annealed for 24 h, and cooled down at a rate of −0.5 K ⋅ min^−1^ to room temperature. The orange‐colored powder was isolated by washing with methanol and stored under argon.

**Crystal structure determination**: Diffraction data were collected at 100(1) K with a four‐circle diffractometer Kappa Apex2 (Bruker) equipped with a CCD detector by using graphite‐monochromated Mo_Kα_ radiation (*λ*=71.073 pm). The raw data were corrected for background, Lorentz and polarization factors,^[45]^ and multiscan absorption correction was applied.^[46]^ The structure was solved using intrinsic phasing in the ShelXT program.^[47]^ Structure refinement against *F* ^2^ with ShelXL^[48]^ included anisotropic displacement parameters for all atoms. As the freely refined deficiency of tellurium matched the surplus of potassium within the standard deviations, the occupancies of Te and K2 were coupled to assure charge balance. The graphical representations of the structure were developed with Diamond.^[49]^ Tables S1 to S3 of the Supporting Information contain crystallographic data.

Deposition Number https://www.ccdc.cam.ac.uk/services/structures?id=doi:10.1002/chem.2021024642062610 (for K_12+6*x*_Fe_6_Te_4−*x*_O_27_) contains the supplementary crystallographic data for this paper. These data are provided free of charge by the joint Cambridge Crystallographic Data Centre and Fachinformationszentrum Karlsruhe http://www.ccdc.cam.ac.uk/structuresAccess Structures service.

**Powder X‐ray diffraction**: X‐ray powder diffraction for phase identification and Rietveld refinement were performed at room temperature on an Empyrean diffractometer (Panalytical) equipped with a curved Ge(111) monochromator using Cu${}_{K\alpha {}_{1}}$ radiation (*λ*=154.056 pm). The program package Topas‐Academics v.5 was used for Rietveld refinement.^[50]^

**Neutron powder diffraction**: Neutron diffraction data of a K_12+6*x*_Fe_6_Te_4−*x*_O_27_ powder sample were measured at the SPODI high‐resolution powder diffractometer at the research reactor Heinz‐Maier‐Leibnitz (FRM II) in Munich. Diffraction data were collected at 4 and 300 K using a closed‐cycle cryostat and a Ge(551) monochromator with a neutron wavelength of *λ*=154.83 pm and a step width of 0.05. The detector bank consists of 80 spatially resolved ^3^He counter tubes (active measuring height: 300 mm; 2 angular range).[^51^, ^52^] The refinement of the nuclear and magnetic structure was done with Jana2006.

**Impedance spectroscopy**: Crystals of K_12+6*x*_Fe_6_Te_4−*x*_O_27_ were ground using a planetary ball‐mill and pressed into pellets with a diameter of 10 mm and a thickness of about 1 mm. Silver paste was used for contacting. The ion conductivity was measured with the AC impedance method using a VMP‐3 (Biologic) potentiostat. The data were collected in the range between 100 Hz and 1 MHz with an applied AC voltage of 10 mV. The impedance of K_12+6*x*_Fe_6_Te_4−*x*_O_27_ was determined, by a circle fit or by fitting the linear part. The ion conductivity was calculated by dividing the thickness of the pellet by the surface area and the impedance.

**Piezo‐response force microscopy**: PFM images were recorded on a SPM1000 (Aist‐NT) with chromium‐platinum coated ElectriMulti75‐G (Budget Sensors) Silicon probes. The probes force constant was 3 N m^−1^, its resonance frequency 75 kHz, and its tip radius was <25 nm. The tip was lowered onto the center of one of the crystals facets. The frequency sweeps all occurred at the same position. Afterwards the surrounding area was scanned by taking a PFM image. For comparison, similar measurements were conducted on piece of z‐cut lithium niobate, which has a known piezoelectric effect, as well as a microscopy glass slide, which features no apparent piezoelectric effect, and hence allows to judge the magnitude of mechanical background response not originating from the sample.

**Magnetic measurements**: The magnetization of a K_12+6*x*_Fe_6_Te_4−*x*_O_27_ powder was analyzed in a SQUID magnetometer MPMS3 (Quantum Design) in vibration sample magnetometer (VSM) mode in the temperature range 2–400 K.

**SEM and EDX analysis**: Scanning electron microscopy (SEM) was performed using a SU8020 (Hitachi) with a triple detector system for secondary and low‐energy backscattered electrons (*U*_a_=5 kV). The composition of selected single crystals was determined by semi‐quantitative energy dispersive X‐ray analysis (*U*_a_=15 kV) using a Silicon Drift Detector (SDD) X‐Max^N^ (Oxford Instruments). The data were processed with the AZtec software package (Oxford Instruments, 2013).

## Conflict of interest

The authors declare no conflict of interest.

## Supporting information

As a service to our authors and readers, this journal provides supporting information supplied by the authors. Such materials are peer reviewed and may be re‐organized for online delivery, but are not copy‐edited or typeset. Technical support issues arising from supporting information (other than missing files) should be addressed to the authors.

Supporting Information
