# Supplementary material for: Potassium Ion Conductivity in the Cubic Labyrinth of a Piezoelectric, Antiferromagnetic Oxoferrate(III) Tellurate(VI)
Source: Chemistry. 2021 Sep 3;27(57):14299–306. doi: 10.1002/chem.202102464 (PMC8596643; doi:10.1002/chem.202102464)
Supplement: Supplementary file 1 — Supporting Information [file CHEM-27-14299-s001.pdf]

Chemistry–A European Journal

Supporting Information

Potassium Ion Conductivity in the Cubic Labyrinth of a Piezoelectric, Antiferromagnetic Oxoferrate(III) Tellurate(VI)

Ralf Albrecht, Markus Hoelzel, Henrik Beccard, Michael Rüsing, Lukas Eng, Thomas Doert, and Michael Ruck*

Content

Synthesis 2
Single-crystal data 5
Chemical Decomposition Experiments 8
Ion conductivity 10
Piezoresponse Force Microscopy 12
Magnetic Properties 13
Reference 14

Synthesis

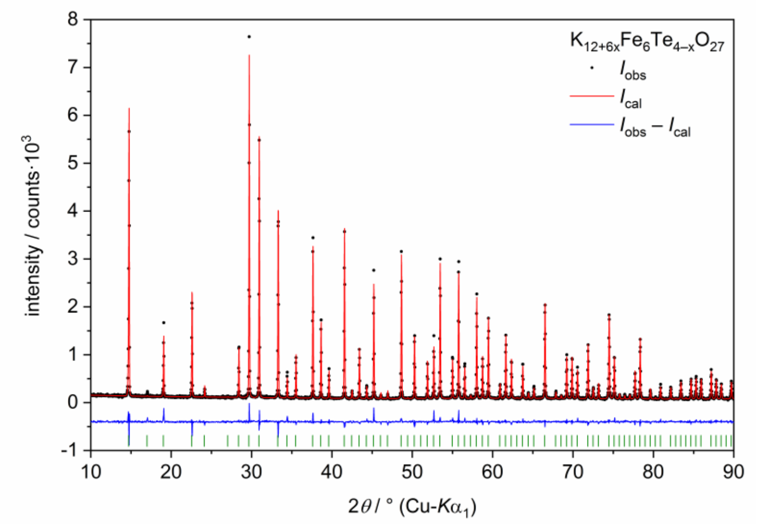

Figure S1. Powder X-ray diffractogram and Rietveld refinement of $K_{12+6x}Fe_6Te_{4-x}O_{27}$ in $I\bar{4}3d$ measured with Cu-Kα_1 radiation (R_p = 5.82 %, wR_p = 7.60 %, gof = 1.07).

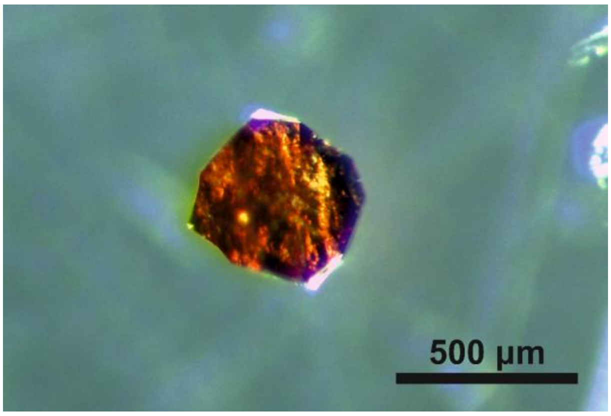

Figure S2. Photograph of a large crystal of $K_{12+6x}Fe_6Te_{4-x}O_{27}$ received by using TeO_2 without H_2O_2 as starting material.

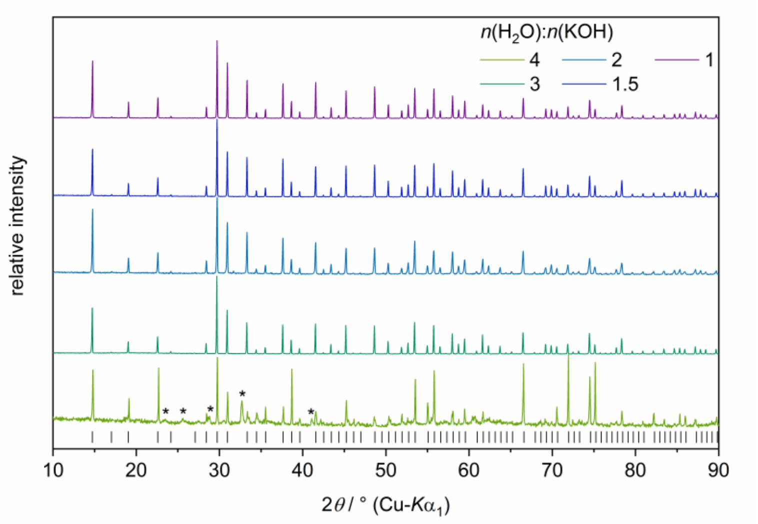

Figure S3. Powder X-ray diffractogram of $K_{12+6x}Fe_6Te_{4-x}O_{27}$ synthesized at different water-base ratios q(K). Reflections marked with an asterisk indicate an unidentified by-product.

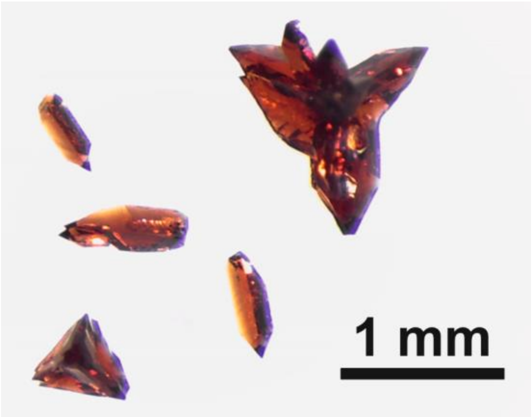

Figure S4. Photograph of $K_{12+6x}Fe_6Te_{4-x}O_{27}$ crystals synthesized with a water-base ratio q(K) = 3.

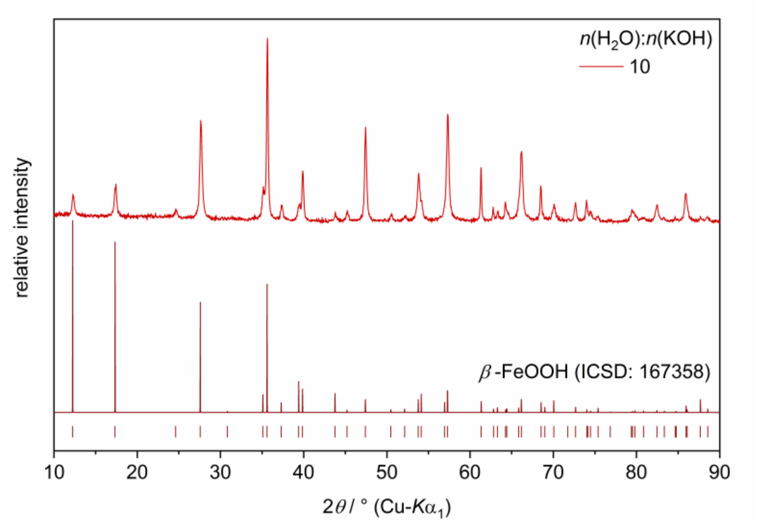

Figure S5. Powder X-ray diffractogram of β-FeOOH synthesized at water-base ratios q(K) = 10.

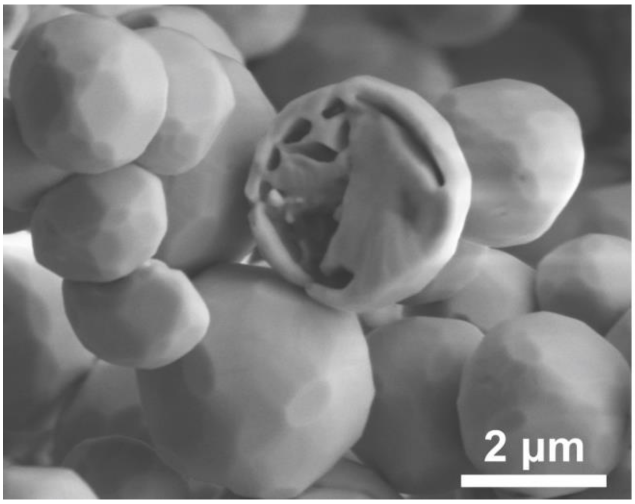

Figure S6. Electron image from spherical crystals of $K_{12+6x}Fe_6Te_{4-x}O_{27}$ received by using $(NH_4)_2TeO_4$ as starting material.

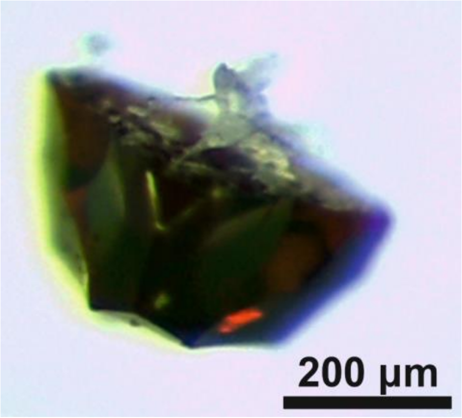

Figure S7. Photograph of a large crystal of $K_{12+6x}Fe_6Te_{4-x}O_{27}$ after one month in air showing thin precipitate crystals on the surface.

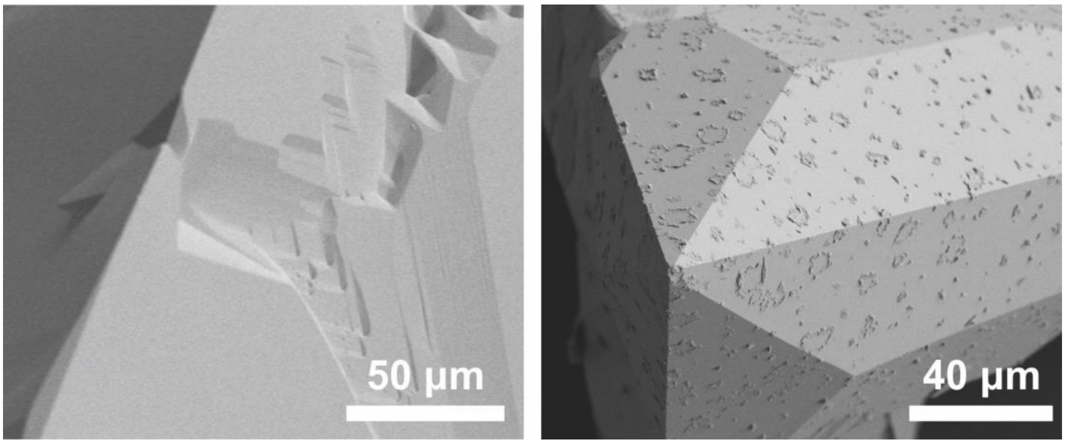

Figure S8. Electron image from crystals of $K_{12+6x}Fe_6Te_{4-x}O_{27}$ directly after the synthesis with TeO_2 (left) and after one week in air (right).

Single-crystal data

Table S1. Atomic coordinates and equivalent isotropic displacement parameters (/ pm²) of $K_{12+6x}Fe_6Te_{4-x}O_{27}$ at 100(1) K.

	x	*y*	*z*	U_{eq}	Occupancy
Te	0.4184(1)	0.4184(1)	0.4184(1)	60(1)	0.944(1)
Fe	0.4994(1)	1/2	1/4	75(1)	1
K1	0.2869(1)	0.3916(1)	0.1961(1)	288(1)	1
K2	0.4702(6)	0.2704(8)	0.1222(6)	411(16)	0.112(2)
O1	0.4464(1)	0.3847(1)	0.2946(1)	106(2)	1
O2	0.4668(1)	0.5340(1)	0.3796(1)	114(2)	1
O3	5/8	1/2	1/4	125(4)	1

Table S2. Anisotropic displacement parameter (/ pm²) of $K_{12+6x}Fe_6Te_{4-x}O_{27}$ at 100(1) K.

	U_{11}	U_{22}	U_{33}	U_{12}	U_{13}	U_{23}
Te	60(1)	60(1)	60(1)	4(1)	4(1)	4(1)
Fe	59(1)	88(1)	79(2)	17(1)	0	0
K1	498(3)	178(2)	187(2)	53(1)	–133(2)	–150(2)
K2	420(50)	390(50)	430(30)	–30(30)	10(30)	–10(20)
O1	128(4)	95(4)	93(4)	–6(3)	12(3)	–11(3)
O2	143(5)	90(4)	108(4)	1(3)	15(3)	–15(3)
O3	68(7)	154(6)	154(6)	0	0	0

Table S3. Crystal data and structure refinement of $K_{12+6x}Fe_6Te_{4-x}O_{27}$.

Crystal system	cubic
Space group (no.), Z	$I\bar{4}3d$ (220),8
Temperature	100(1) K
Wavelength	71.073 pm
Lattice parameters	*a* = 1474.4(1) pm
Unit cell volume	3205.1(7) 10^6 pm³
Density (calc.)	3.66 g·cm^{-3}
No. of reflections / parameters	2180 / 51
$2\theta_{max}$ (MoK_α)	90°
R_{int}, R_σ	0.043, 0.012
$R_1[F>2\sigma(F)]$, $wR_2(F^2)$	0.014, 0.033
GooF(F^2)	1.049
Residual electron density	–1.02 to 0.99 e 10^{-6}·pm^{-3}

Table S4: EDX results on a $K_{12+6x}Fe_6Te_{4-x}O_{27}$ crystal. The low potassium content is caused by the preparation of the epoxy puck, as water and sandpaper had to be used to achieve a plain surface.

	K	Fe	Te	O
theoretical	12	6	4	27
measured	10.9(1)	6.0(1)	3.8(1)	33(1)

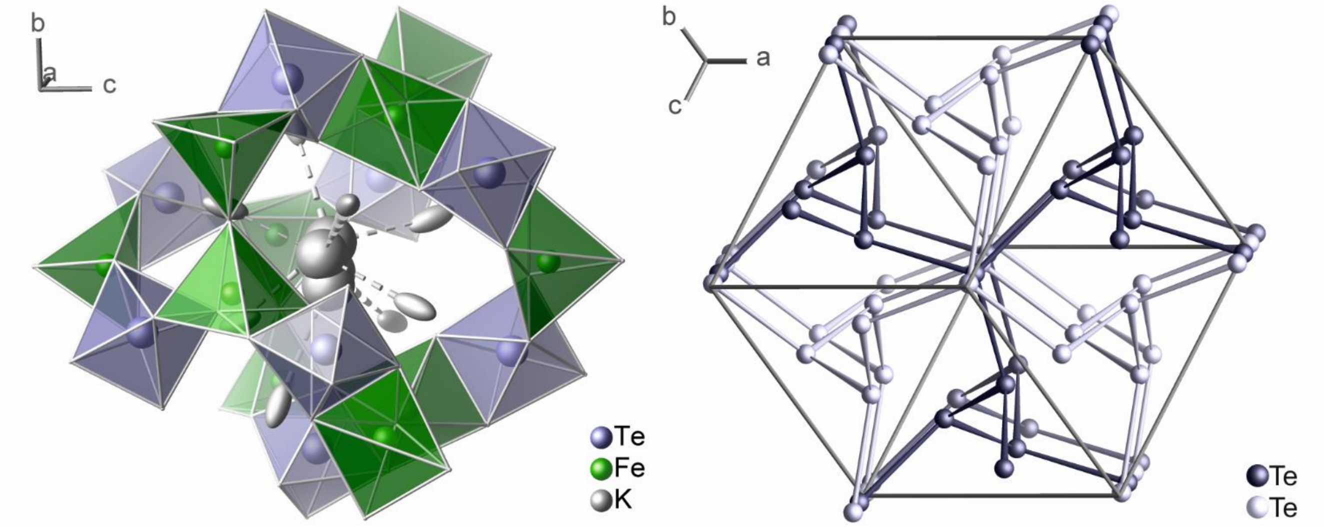

Figure S9. Left: Surrounding of the potassium node within the ferrate tellurate framework in $K_{12+6x}Fe_6Te_{4-x}O_{27}$. Right: The two separated tellurium networks form helices (Te–Te = 552.2(1) pm) with opposite chirality.

In the manuscript, we report about single-crystal X-ray diffraction experiments with the aim to analyze the decomposition process. In in the case of a non-aged single-crystal, weak reflections ($I/\sigma(I)$ ratio of about 1) violating the reflection conditions of the space group $I\bar{4}3d$ were found in the diffraction pattern (Figure A). By analyzing the reflection data in detail, we are convinced that all violating reflections are caused by Renninger or $\lambda/2$ effects. The Renninger effect is present when several reflections have the same Laue conditions resulting in multiple reflection (*Umweganregung*).[1] Those reflections always occur within the Bravais lattice, but can violate zonal or serial reflection conditions of the respective space group. However, Renninger reflections are only present under special geometrical positions, i.e. at a slightly different measuring position the reflection will vanish. Hence, multiple measurements of the identical reflections can lead to very different intensities (after applying all corrections). The Renninger effect is typically visible with large crystals with a high quality (low mosaic spread).

The data set (R_σ = 0.6 %) of the non-aged single-crystal of $K_{12+6x}Fe_6Te_{4-x}O_{27}$ has a high redundancy (>100) and a large overall mean $I/\sigma(I)$) ratio of about 170. When considering reflection that do not fulfil the reflection conditions of $I\bar{4}3d$, which have low intense corresponding 2*h* 2*k* 2*l* reflections, the majority of these reflections have intensities below $I_o \geq 4\ \sigma(I_o)$, however, few have significant higher intensities caused by the Renninger effect (Figure B).

Some of the reflections violating the reflection conditions of $I\bar{4}3d$ have $I_o \geq 3\sigma(I_o)$ for all measured data, e.g. {0 0 2}, which is very unlikely for the Renninger effect. We suppose that these reflections are caused by the λ/2 effect, since they are found at small diffraction angles and their corresponding 2*h* 2*k* 2*l* reflections are very strong. For example, the largest deviations $(F_o–F_c)/\sigma(F_o)$ is positive and found for the reflection {0 2 4}, while the strongest reflection in the data set is the reflection {0 4 8}.

Similar effects were observed in the diffraction data sets of the single-crystal that had been aged for one and two weeks, but to a lesser extent as the partial decomposition of the single-crystal reduced its crystallinity.

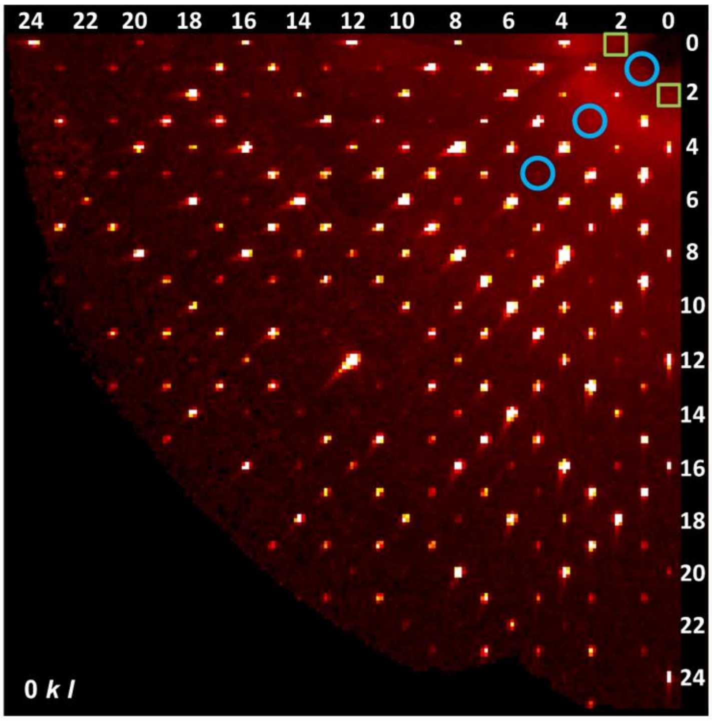

Figure A. Reconstructed 0kl plane of $K_{12+6x}Fe_6Te_{4-x}O_{27}$ measured directly after the synthesis. Note the highlighted reflections, which do not fulfil the reflection conditions of $I\bar{4}3d$.

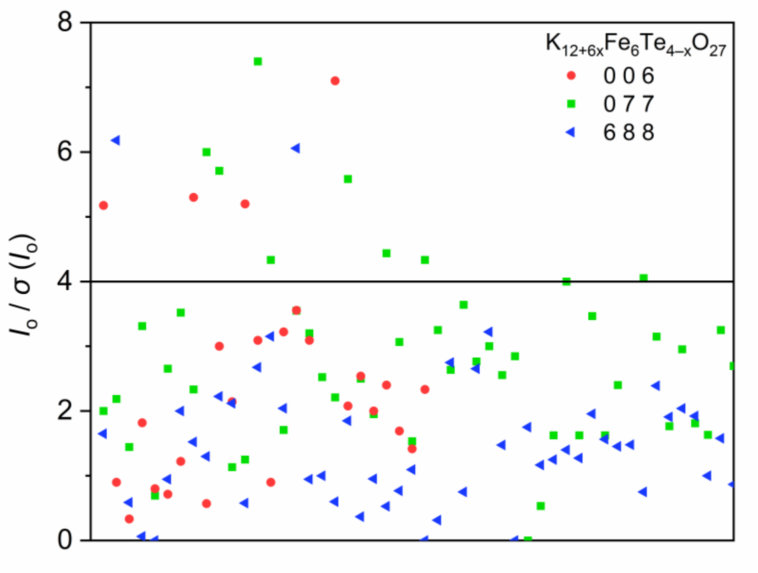

Figure B. Ratio of the X-ray diffraction intensity and the standard deviation of selected reflection sets of non-aged $K_{12+6x}Fe_6Te_{4-x}O_{27}$ single-crystal. Only a small fraction of a reflection set have an unusually large I/σ ratio caused by the Renninger effect.

Chemical Decomposition Experiments

Table S5. Single-crystal structure date of $K_{12+6x}Fe_6Te_{4-x}O_{27}$ measured directly after the synthesis and subsequent ageing in air.

$K_{12+6x}Fe_6Te_{4-x}O_{27}$	after synthesis	aged for one week	aged for two weeks	aged for three weeks	aged for four weeks	aged for five weeks
Crystal system	cubic	cubic	cubic	cubic	cubic	cubic
Space group (no.)	$I\bar{4}3d$ (220)	$I\bar{4}3d$ (220)	$I\bar{4}3d$ (220)	$I\bar{4}3d$ (220)	$I\bar{4}3d$ (220)	$I\bar{4}3d$ (220)
Temperature / K	298(1)	298(1)	298(1)	298(1)	298(1)	298(1)
Lattice parameter / pm	1475.31(4)	1474.95(4)	1474.42(5)	1474.58(5)	1474.30(8)	1473.45(10)
Volume / (10^6 pm³)	3211.1(3)	3208.7(3)	3205.3(3)	3206.3(3)	3204.5(5)	3198.9(7)
Density (calc.) / (g cm^{-3})	3.671	3.650	3.667	3.663	3.660	3.664
$2\theta_{max}$ / °	75	75	75	75	75	75
No. of measured reflections	88350	93668	84322	84463	88607	68938
No. of independent reflections	1421	1418	1418	1421	1419	1419
No. of parameters	50	49	50	50	51	51
Mean $I/\sigma(I)$	169	133	115	83	24	24
Redundancy	108	115	104	104	109	85
R_{int}, R_σ	0.027, 0.006	0.036, 0.008	0.043, 0.010	0.061, 0.014	0.211, 0.053	0.203, 0.056
$R_1[F_o > 2\sigma(F_o)]$, $wR_2(F_o^2)$	0.015, 0.035	0.014, 0.031	0.014, 0.031	0.016, 0.035	0.037, 0.080	0.036, 0.077
GooF (F^2)	1.248	1.219	1.145	1.128	1.062	1.038
Residual electron density / (e 10^{-6} pm^{-3})	0.83 to −0.68	0.68 to −0.78	0.66 to −0.68	0.62 to −0.71	0.90 to −0.92	0.81 to −0.88

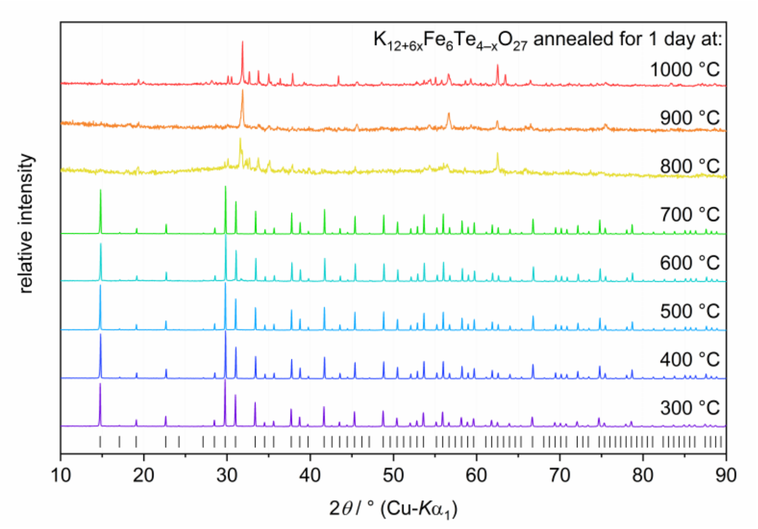

Figure S10. Powder diffraction pattern of $K_{12+6x}Fe_6Te_{4-x}O_{27}$ samples annealed at different temperatures for one day. The tick marks represent the calculated reflection positions of $K_{12+6x}Fe_6Te_{4-x}O_{27}$.

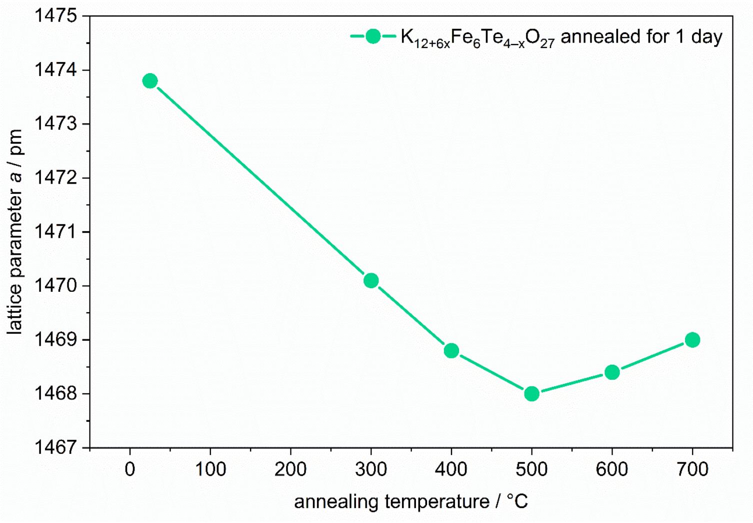

Figure S11. Representation of the lattice parameter *a* determined by Le Bail fitting powder diffraction patterns of $K_{12+6x}Fe_6Te_{4-x}O_{27}$ samples annealed at various temperatures in comparison with the one measured directly after the synthesis. The calculated standard deviations are smaller than the plotted symbol.

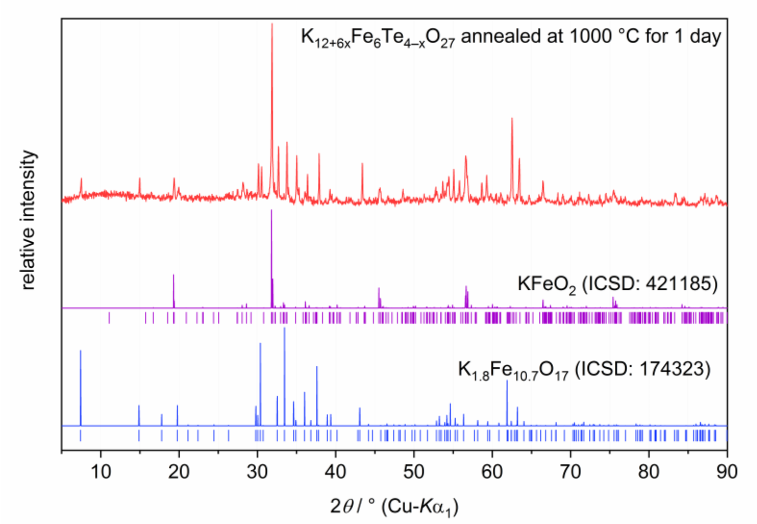

Figure S12. Powder diffraction pattern of $K_{12+6x}Fe_6Te_{4-x}O_{27}$ annealed at 1000 °C for one day.

Ion conductivity

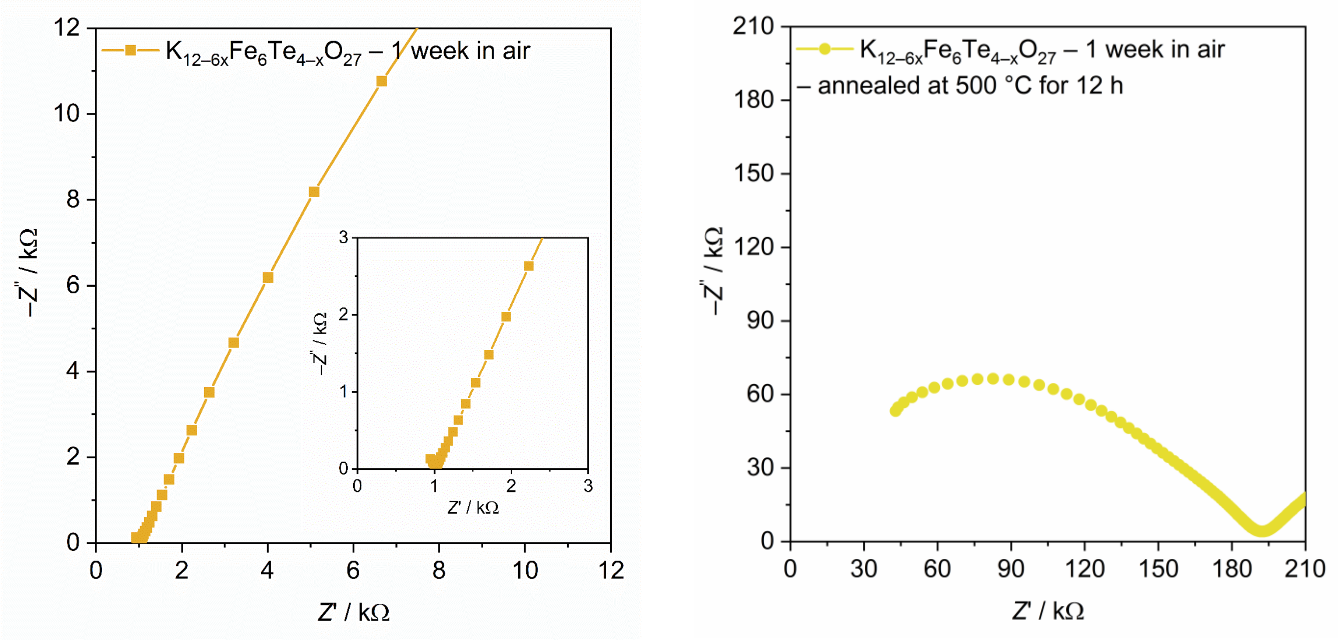

Figure S13. Impedance spectra of $K_{12+6x}Fe_6Te_{4–x}O_{27}$ pellets after storing the powder one week in air (left) and subsequent annealing at 500 °C for 12 h (right) measured at room temperature. In these impedance spectra, a frequency range from 1 MHz to 10 Hz (1 Hz for the right figure) is presented.

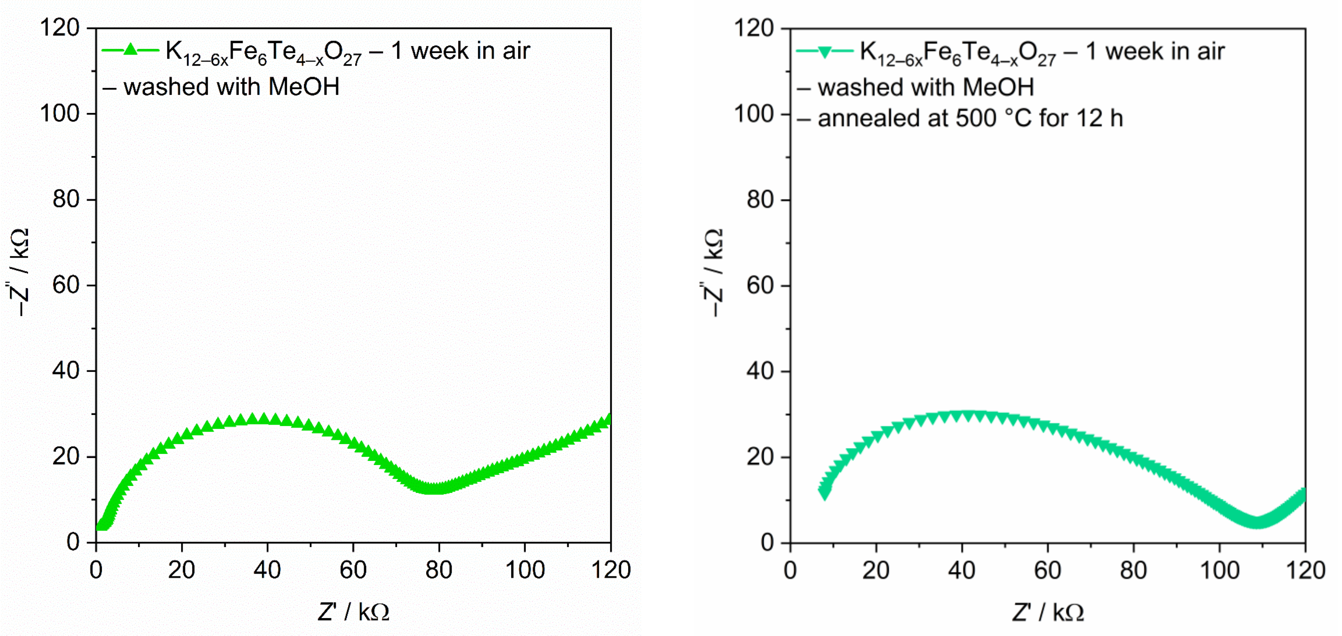

Figure S14. Impedance spectra of $K_{12+6x}Fe_6Te_{4–x}O_{27}$ pellets after washing the aged powder with methanol (left) and subsequent annealing at 500 °C for 12 h (right) measured at room temperature. In these impedance spectra, a frequency range from 1 MHz to 10 Hz (1 Hz for the right figure) is presented.

Table S6. The ion conductivity of $K_{12+6x}Fe_6Te_{4-x}O_{27}$ received from the impedance measurements at room temperature. The impedance of the samples was determined, if possible, by a circle fit or, if not, by fitting the linear part. The ion conductivity was calculated by dividing the thickness of the pellet with the impedance and the surface area.

Ion conductivity	$K_{12+6x}Fe_6Te_{4-x}O_{27}$
after ball-milling / (S·cm^{-1})	2.3 10^{-6}
after ball-milling and storing one day in air / (S·cm^{-1})	1.7 10^{-4}
after ball-milling and storing one week in air / (S·cm^{-1})	2.3 10^{-4}
aged powder (one week) annealing at 500 °C for 12 h / (S·cm^{-1})	1.2 10^{-6}
aged powder (one week) washed with methanol / (S·cm^{-1})	8 10^{-8}
aged powder (one week) washed with methanol and subsequent annealing at 500 °C for 12 h / (S·cm^{-1})	6 10^{-7}

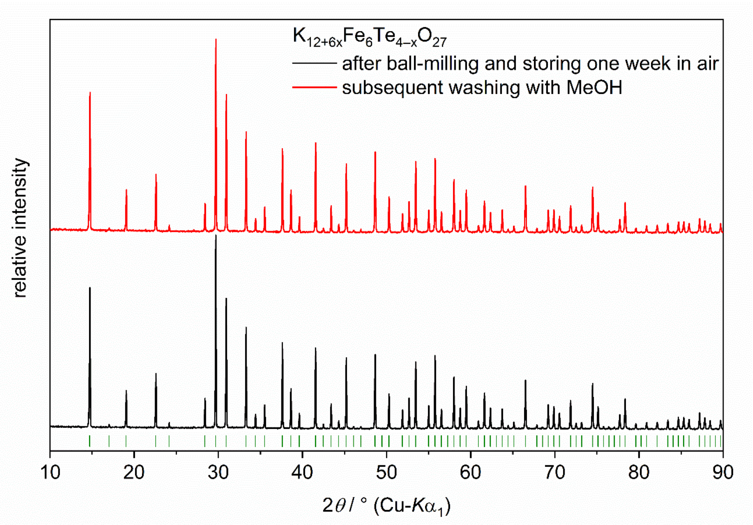

Figure S15. Powder diffraction pattern of $K_{12+6x}Fe_6Te_{4-x}O_{27}$ samples after ball-milling and storing one week in air and subsequent washing with methanol.

Piezoresponse Force Microscopy

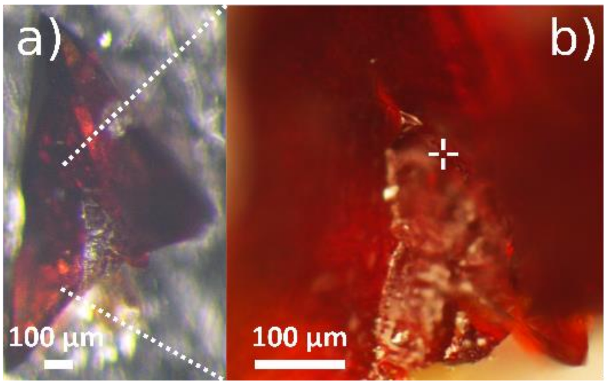

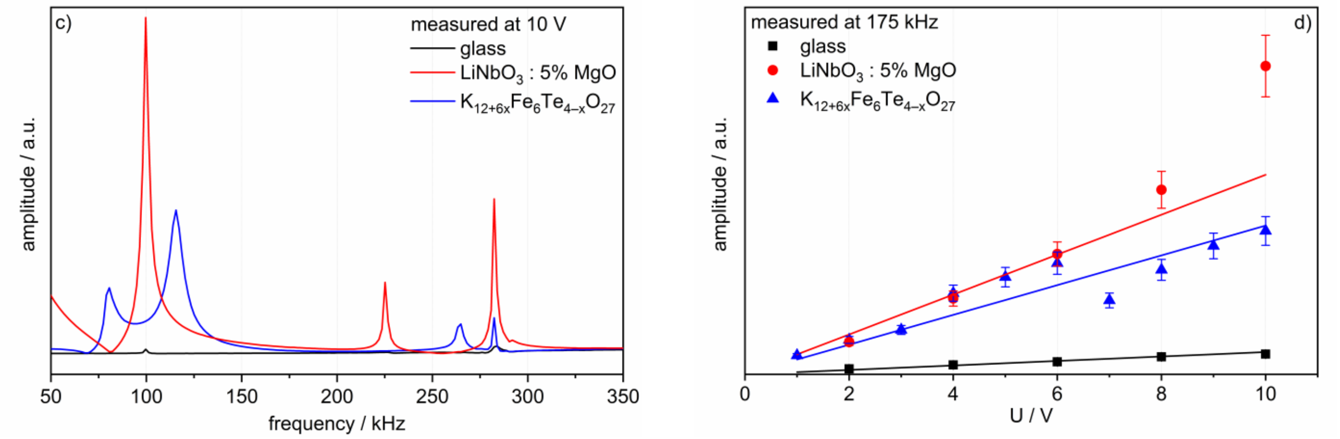

Figure S16. Optical image of a selected $K_{12+6x}Fe_6Te_{4-x}O_{27}$ crystal (a) with the spot of the measurement (b). The piezoelectric response of $K_{12+6x}Fe_6Te_{4-x}O_{27}$ was measured dependent on the AC-frequency and compared with *z*-cut $LiNbO_3$ and glass (c). In addition, the piezoelectric amplitude depending on the voltage was measured at a frequency of 175 kHz (d).

Magnetic Properties

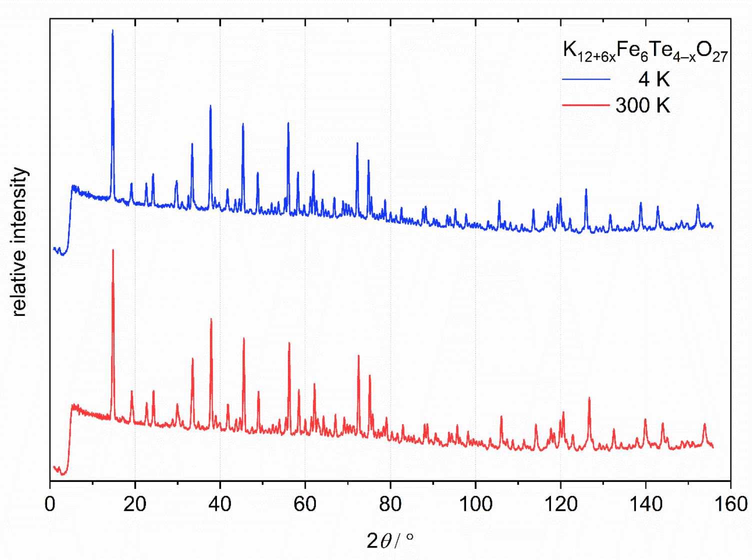

Figure S17. Neutron diffraction patterns (λ = 154.83 pm) of $K_{12+6x}Fe_6Te_{4-x}O_{27}$ measured at two different temperatures.

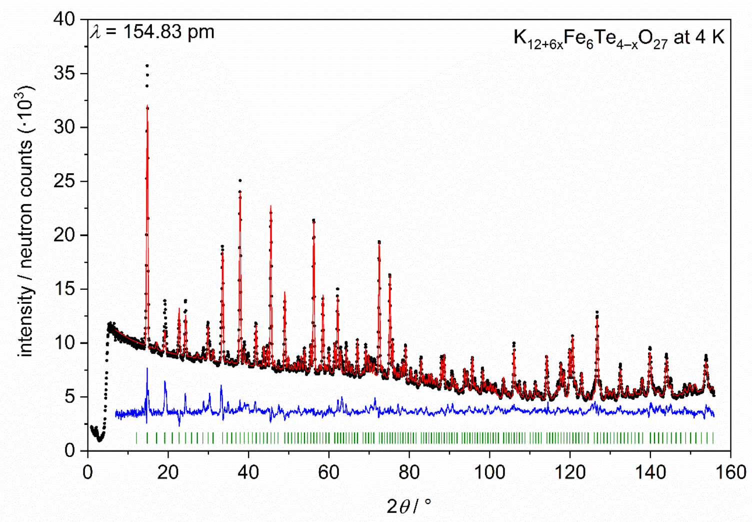

Figure S18. Rietveld refinement of the neutron diffraction pattern of $K_{12+6x}Fe_6Te_{4-x}O_{27}$ in $I\bar{4}'3d'$ measured at 4 K (a = 1469.03(1) pm, R_p = 7.12 %, wR_p = 6.53 %, gof = 2.80).

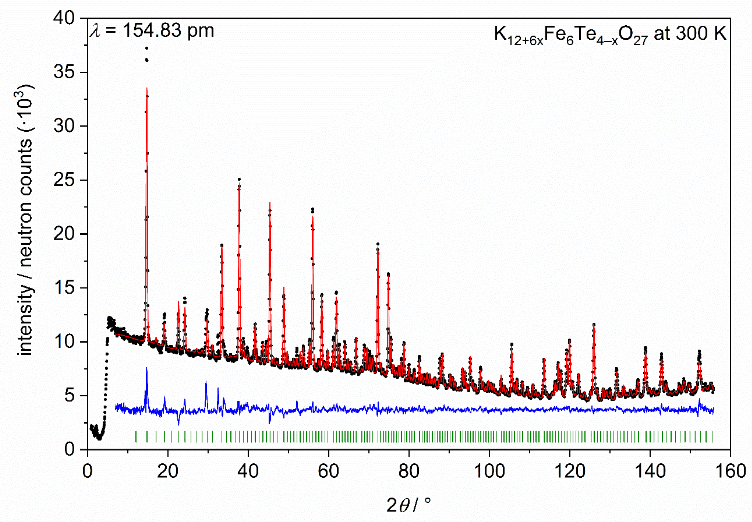

Figure S19. Rietveld refinement of the neutron diffraction pattern of $K_{12+6x}Fe_6Te_{4-x}O_{27}$ in $I\bar{4}'3d'$ measured at 300 K (a = 1473.95(1) pm, R_p = 4.81 %, wR_p = 4.68 %, gof = 1.99).

Reference

[1] M. Renninger, *Z. Physik* **1937**, *106*, 141–176.
